# Repetitive transcranial magnetic stimulation in patients with fibromyalgia: A protocol for systematic review and network meta-analysis

**DOI:** 10.1097/MD.0000000000031696

**Published:** 2022-11-25

**Authors:** Yangyang Wang, Junfang Lei, Hong Qiao, Jiqin Tang, Xiaohua Fan

**Affiliations:** a College of Rehabilitation Medicine, Weifang Medical University, Weifang, Shandong, China; b School of Rehabilitation Medicine, Shandong University of Traditional Chinese Medicine, Jinan, Shandong, China; c Department of Rehabilitation, Shandong Provincial Hospital, Jinan, Shandong, China.

**Keywords:** fibromyalgia, network meta-analysis, pain, repetitive transcranial magnetic stimulation

## Abstract

Fibromyalgia is a chronic disease characterized by widespread pain. Repetitive transcranial magnetic stimulation (rTMS) effectively relieves pain intensity in patients with fibromyalgia. The frequency and target site of rTMS have significant roles in therapy effectiveness. However, there is disagreement over the best rTMS protocol. Thus, we will conduct a thorough systematic review and network meta-analysis to rank the efficacy of these various rTMS protocols and determine which is most beneficial in lowering pain and enhancing the quality of life.

**Methods:**

Databases PubMed, Web of Science, Embase, and Cochrane Library will be searched for clinical randomized controlled trials of rTMS in fibromyalgia. The retrieval time is from the inception of the database until October 1, 2022. Following the Cochrane Handbook, 2 reviewers will independently review the literature, extract data, and evaluate the risk of bias of included articles. Pain intensity and quality of daily life are outcome indicators. Stata 17.0 and ADDIS 1.16.8 software will be used for pairwise meta-analysis and network analysis to evaluate the effectiveness of rTMS and the ranking probability of all protocols. The recommended grading assessment, development, and evaluation will be used to assess the overall quality of the evidence.

**Results:**

The meta-analysis and probability ranking of the network determined the best TMS protocol for fibromyalgia.

**Conclusion:**

This study will provide systematic support of evidence-based medicine for TMS in fibromyalgia, integrate the results of direct and indirect comparisons of the efficacy of different rTMS protocol, and provide the best one.

## 1. Introduction

Fibromyalgia is a chronic complex disease that is characterized by widespread or regional musculoskeletal pain along with fatigue, sleep disturbances, mood disorders, and cognitive impairment.^[1,2]^ These symptoms seriously degrade a patient’s psychological state, quality of life, and financial situation.^[3]^ In the general population, the incidence ranges from 0.2% to 6.6%.^[4]^ The pathophysiology of fibromyalgia is currently unknown, but central sensitization is typically used to account for abnormal pain perception.^[5]^ The patients have a hyperexcitable central nervous system, which causes nociceptive hypersensitivity and pain in response to typical non-noxious stimuli (such as abnormal pain).^[6–8]^

Repetitive transcranial magnetic stimulation (rTMS), a noninvasive method of neurocentral modulation, has been found to be beneficial in reducing chronic pain.^[9,10]^ In 2020, the European expert group recommendation for fibromyalgia treatment-level B included rTMS.^[11]^ The basic principle of rTMS is to limit the activity of regions that cause pain by modulating cortical excitability.^[12]^ The mechanisms of rTMS also include the enhancement of endogenous opioid release and the generation of neuroplastic alterations in pain pathways.^[12–14]^ Depending on the cortical area stimulated and rTMS setting parameters (especially frequency), rTMS can have various therapeutic effects.^[15]^ Currently, the key brain areas targeted by rTMS in pain are the primary motor cortex (M1) and dorsolateral prefrontal cortex.^[16]^ Moreover, low-frequency (LF) rTMS (≤1 Hz) and high-frequency (HF) rTMS (>1 Hz) can be distinguished based on frequency.^[17]^ The optimum rTMS protocol (i.e., target site/stimulation frequency combination) has been investigated in some conventional meta-analyses.^[18,19]^ Choo et al showed that HF rTMS on the M1 significantly decreased the visual analog scale scores in patients with fibromyalgia by including 10 pertinent randomized controlled trials (RCTs).^[18]^ After the treatment course, analgesic efficacy continued for 1 to 4 weeks. According to Sun,.^[19]^ LF rTMS in the dorsolateral prefrontal cortex appears to be the most effective treatment for pain. Various analyses have produced different results. However, it is still unclear whether to stimulate the dorsolateral prefrontal cortex or M1 region using HF or LF rTMS. Moreover, the aforementioned studies were traditionally paired meta-analyses; thus, a network meta-analysis (NMA) would be more appropriate to evaluate the effectiveness of various combination protocols.^[20]^ NMA enables direct and indirect comparisons of all rTMS protocols, analyses of their effectiveness in fibromyalgia reduction, and ranks the effects of parameter combinations.^[21]^ Compared with conventional meta-analyses, NMA has greater utility.^[22]^ To the best of our knowledge, no NMA has been conducted on this important subject. Thus, we aim to perform a comprehensive NMA to compare the efficacy of several rTMS protocols in patients with fibromyalgia.

## 2. Methods

This systematic review and NMA followed the guidelines of the Preferred Reporting Items for Systematic Review and Meta-Analysis Protocols (PRISMA-P).^[23]^ and the PRISMA extension statement for Reporting of Systematic Reviews Incorporating Network Meta-analysis of health care interventions (PRISMA-NMA).^[24]^ And it was registered with PROSPERO, number 42022350378.

### 2.1. Eligibility criteria

We focused on published, peer-reviewed randomized controlled trials, and developed the following criteria based on the Patient, Intervention, Comparison, and Outcome framework.

#### 2.1.1. *Type of participant*.

Participants (aged ≥ 18 years) were diagnosed with fibromyalgia according to American College of Rheumatology criteria or other standard criteria.^[1,25]^

#### 2.1.2. *Type of interventions*.

The experimental group received rTMS. If it is a multigroup study, only the groups that meet the requirements of this study will be selected.

#### 2.1.3. *Type of comparators*.

Comparators will be sham interventions, or blank control, or comparison of active forms of rTMS.

#### 2.1.4. *Outcomes*.

The predetermined primary indicators is pain measured by Numeric Rating Scale, Visual Analogue Scale, widespread pain index, the McGill Pain Questionnaire, and so on. The quality of life will be included as a secondary outcome.

#### 2.1.5. *Exclusion criteria*.

1. Reviews or systematic reviews, conference abstracts, case reports, and non-RCTs;
2. Patients with other diseases that cause pain;
3. The outcome index of the study was not reported, or the evaluation index of the curative effect was not standard or unclear.

### 2.2. *Information sources*

The PubMed, Embase, Web of Science, and Cochrane Library were searched, from their inception until September 16, 2022. Medical Subject Headings and free-text search terms related to “repetitive transcranial magnetic stimulation,” and “fibromyalgia” were used. Taking Embase as an example, the detailed search strategy is as follows: (“fibromyalgia”/exp OR fibromyalgias:ti,ab,kw OR “muscular rheumatism”:ti,ab,kw OR fibrositis:ti,ab,kw OR fibrositides:ti,ab,kw OR “diffuse myofascial pain syndrome”:ti,ab,kw OR “fibrositic nodule”:ti,ab,kw OR “fibrositis syndrome”:ti,ab,kw OR myalgia:ti,ab,kw OR fibro:ti,ab,kw) AND (“transcranial magnetic stimulation”/exp OR “repetitive transcranial magnetic stimulation”/exp OR “transcranial magnetic stimulations”:ti,ab,kw OR tms:ti,ab,kw OR rtms:ti,ab,kw).^[26]^ The strategies of other databases can be adjusted according to their requirements. There were no limitations on language. In addition, the reference lists of included studies and previously published systematic reviews were hand-searched to ensure that all relevant literature was identified.

### 2.3. Selection of studies

**Figure 1.**
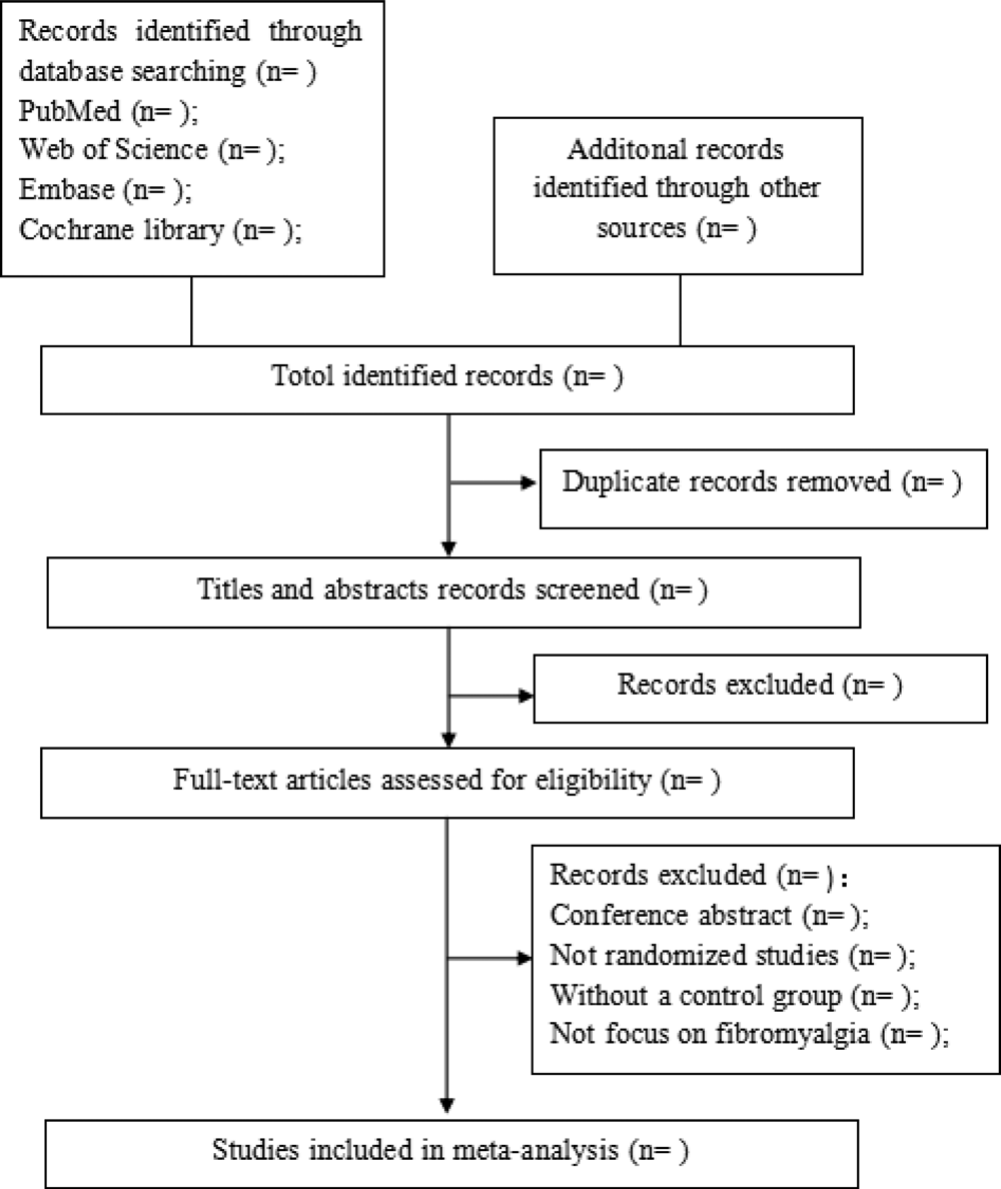
Flow diagram of the study.

First, the retrieval results from the 4 databases will be imported into EndnoteX8 for merging to establish an information database, and the software is used to remove duplicates. Second, we excluded reviews, conference papers, animal experiments, and other literature that did not meet the inclusion criteria by reading the literature title and abstract. Finally, by reading the full text, we screened out the RCTs related to fibromyalgia with rTMS. During the screening process, it is necessary to record in detail the exclusion reasons that are not included in the literature. A third party was consulted to decide on inclusion in case of differences and disputes. The specific screening process of this study is shown in Figure 1.

### 2.4. Data extraction and management

The data will be extracted according to a preformulated table, including the following aspects.

1. Published details: the first author, time of publication, country, and type of study;
2. Basic information of the patients: age, sex, sample size, diagnostic criteria, and course of the disease;
3. Intervention/control measures: type of rTMS, frequency and intensity of stimulation; stimulation site; pulse number, coil type, frequency and duration of treatment, and setting of the control group;
4. Outcome: scores on assessment scales related to pain and quality of life.

### 2.5. Study quality evaluation and quality of evidence

Two reviewers independently used the Cochrane Risk of Bias tool V.2 (RoB 2) to assess risk bias in the included literature.^[27]^ The tool includes 5 domains of potential introduction of bias: randomization, deviation from intended intervention, missing outcome data, outcome measures, and reporting bias. The authors judged the risk of bias for each domain as “low risk of bias,” “some concerns,” or “high risk of bias” through one or more signaling questions. These projects help to determine whether there are sources of methodological bias in the study.

The grading of recommendations, assessment, development, and evaluation (GRADE) will be used to assess the quality of the direct and indirect evidence in this study.^[28]^ Two reviewers independently evaluate the following 5 areas: risk of bias, inconsistency, imprecision, indirectness, and risk of publication bias. The quality of evidence was graded on a scale of “very low,” “low,” “moderate,” and “high.”

### 2.6. Data synthesis and statistical methods

#### 2.6.1. *Pairwise and NMA*.

We will use Stata 17.0 software to conduct a traditional meta-analysis of the included outcome indicators. All the included outcome indices are continuous variables, so the weighted mean difference and standardized mean difference will be used as the effect size. The estimated value and confidence interval of the effect size are provided. The significance threshold was set to *P* < .05.^[29]^

We will use the Stata 17.0 software to draw the network diagram. The network diagram consists of points and lines. The point represents an intervention, and the size of the point represents the sample size that accepts the measure. The larger the point, the larger the sample size, and vice versa. The connections between points represent a direct or indirect comparison between the 2 interventions. The thickness of the edge represents the number of studies; the thicker the edge is, the greater the number of studies, and vice versa. The ADDIS 1.16.8 starts the NETMETA program, calls the data results of the random-effects model based on the Bayesian Markov chain Monte (MCMC Carlo) algorithm, and evaluates and processes it. Convergence is evaluated according to the final iteration effect, which is the potential scale reduction factor. The closer the potential scale reduction factor value is to 1, the better is the convergence effect, and the higher the reliability of the results obtained by the consistency model analysis. We will analyze the results of all direct or indirect comparisons and estimate the rank probability of each group according to the MCMC method to evaluate which TMS modality has the best effect on fibromyalgia.^[30]^

#### 2.6.2. *Assessment of heterogeneity*.

In traditional meta-analyses for paired comparisons, we will carry out a subgroup analysis according to the heterogeneity factor using the χ² test. *I*² is selected as the statistic of heterogeneity among studies in the quantitative subgroup. If *P* ≥ .1 and *I*^2^ ≤ 50%, the heterogeneity between studies is considered acceptable, and a fixed effect model will be used for meta-analysis; if *P* < .1 and *I*^2^ > 50%, there is high heterogeneity among the included studies, and sensitivity or subgroup analyses will be performed to identify sources of heterogeneity. A random-effects model will be used for analysis.

#### 2.6.3. *Subgroup and sensitivity analyses*.

When the meta-analysis results are positive and more than 3 articles are included, a sensitivity analysis will be performed. The included studies shall be excluded one by one, and the meta-analysis shall be performed again to observe the excluded meta-results. If the meta-result remain positive after elimination, the meta-result will be considered stable; otherwise, the meta-result is considered to be unstable.

When there is high heterogeneity in the results of the meta-analysis, a subgroup analysis will be conducted according to the patient’s age, sex, course of disease, treatment cycle, and research quality to analyze the possible sources of heterogeneity.

#### 2.6.4. *Assessment of inconsistency*.

The node-splitting analysis method evaluates the consistency between indirect comparison and direct comparison. When *P* > .05, it will be considered that there is no statistical inconsistency between the 2 results, and the consistency model will be used for analysis. In contrast, if it is considered that there is inconsistency between the 2 comparison results, the inconsistent model will be used for analysis.^[31]^

#### 2.6.5. *Publication bias*.

When at least 10 studies are included in the meta-analysis, the funnel plot method will be used to assess potential publication bias. The figure is inverted, funnel-shaped, and symmetrical, indicating that the possibility of publication bias is small. If the data are biased, it means that there is a greater possibility of publication bias.^[32]^

## 3. Discussion

To the best of our knowledge, this will be the first NMA study to evaluate the effectiveness of rTMS protocols for fibromyalgia. Studies using rTMS and other noninvasive neuromodulation techniques for pain are increasing exponentially. However, because rTMS has so various alternative settings for stimulation frequency, duration, intensity, and target site, there has been no agreement on the optimal therapeutic strategy.^[33]^ This has partly hindered the efficacy and normalized application of rTMS. Evidence shows that the target site and stimulation frequency of rTMS are highly linked with its analgesic effects.^[34]^ Therefore, we will focus on the efficacy of different combinations of rTMS frequencies and sites. The MNA will offer relative rankings of various protocols based on direct and indirect comparative evidence and thorough analysis of the research on the benefit of rTMS for fibromyalgia.

This study has some limitations. For instance, even if we can ensure homogeneity between experiments, the parameter settings of rTMS (duration, intensity, etc.) will still differ somewhat. Additionally, publication and selection biases may affect the reliability of the results. However, we still hope that the study will provide the finest selection of TMS protocols and reliable evidence-based medicine for clinical practice.

## Author contributions

**Conceptualization:** Yangyang Wang, Junfang Lei, Jiqin Tang, Xiaohua Fan.

**Data curation:** Yangyang Wang, Hong Qiao, Jiqin Tang.

**Formal analysis:** Yangyang Wang, Junfang Lei, Jiqin Tang.

**Funding acquisition:** Jiqin Tang.

**Investigation:** Yangyang Wang, Jiqin Tang, Xiaohua Fan.

**Methodology:** Yangyang Wang, Junfang Lei, Hong Qiao, Jiqin Tang.

**Project administration:** Jiqin Tang, Xiaohua Fan.

**Resources:** Yangyang Wang, Junfang Lei, Hong Qiao, Jiqin Tang.

**Software:** Yangyang Wang, Junfang Lei, Hong Qiao, Xiaohua Fan.

**Supervision:** Jiqin Tang, Xiaohua Fan.

**Validation:** Jiqin Tang, Xiaohua Fan.

**Visualization:** Yangyang Wang, Jiqin Tang, Xiaohua Fan.

**Writing–original draft:** Yangyang Wang, Junfang Lei, Hong Qiao.

**Writing–review and editing:** Yangyang Wang, Junfang Lei, Hong Qiao, Jiqin Tang, Xiaohua Fan.
